# Nicotine dependence and visceral adiposity as risk factors for the development and severity of carotid artery stenosis

**DOI:** 10.25122/jml-2022-0252

**Published:** 2023-03

**Authors:** Mahmood Shaker Khazaal, Farqad Bader Hamdan, Qasim Sharhan Al-Mayah

**Affiliations:** 1 Department of Physiology, College of Medicine, Al-Nahrain University, Baghdad, Iraq; 2 Medical Research Unit, College of Medicine, Al-Nahrain University, Baghdad, Iraq

**Keywords:** carotid artery stenosis, nicotine dependence, visceral adiposity index, lipid accumulation product, BMI – Body Mass Index, CAS – Carotid Artery Stenosis, CVD – Cardiovascular Disease, DUS – Doppler ultrasound, IHD – Ischemic Heart Disease, LAP – Lipid Accumulation Products, VAI – Visceral Adiposity Index, WC – Waist Circumference, WHR – Weight, Waist-Hip Ratio

## Abstract

Nicotine dependence (ND) and visceral adiposity are emerging as independent risk factors for cardiovascular diseases, including carotid artery stenosis (CAS). This study aimed to determine the relationship between ND and the contribution of abdominal fat to the onset of CAS, which is indicated by a luminal narrowing of at least 60% as determined by duplex and/or Doppler ultrasound. We prospectively collected data from 60 patients with CAS and 60 age- and gender-matched healthy subjects. The Fagerström Test for Nicotine Dependence (FTND), a common research tool, was used in the study. The original questionnaire was designed to gather social and demographic data. Anthropometric measurements, visceral adiposity index (VAI), and lipid accumulation products (LAP) were used to assess obesity. Most patients showed a high or mild-moderate degree of ND: 46.67% and 35%, respectively. The median visceral adiposity index (VAI) and lipid accumulation product (LAP) in patients was 3.92 and 32.83, respectively. Prolonged smoking duration, increased intensity, and high ND are hallmarks of CAS patients.

## INTRODUCTION

Cardiovascular diseases (CVD) have become a leading cause of mortality and early morbidity, with a steadily increasing worldwide prevalence over the past 20 years [1,2]. The distribution of fat, rather than the total amount of adipose tissue, has a greater impact on the outcome and mortality of CVD [3]. Chronic CVD is associated with excessive visceral fat, demonstrating a strong link between cardiometabolic health and an abundance of ectopic fat [4]. This risk factor is independent of general adiposity and is regarded as a major contributor to CVD [5].

The extent and severity of coronary artery plaques and the incidence of major adverse cardiac events are inversely correlated with increased abdominal fat tissue/subcutaneous fat tissue ratio [3,6]. Nevertheless, the impact of obesity on carotid atherosclerosis disease is still under observation [7], and there is limited research on the effect of adipose tissue on carotid artery atherosclerosis and the risk of stenosis [8]. Smoking has long been recognized as a major contributor to clinical CVD and is associated with the development of atherosclerosis [9]. In fact, smoking is a major risk factor for peripheral artery diseases (PAD), with data suggesting that up to half of all cases of PAD can be attributed to smoking [10]. Even those who have quit smoking are still more likely to develop PAD than those who have never smoked [11]. Passive smoking increases the risk of vascular damage and the diagnosis of PAD [12]. Smoking impairs the immune system, promotes proatherogenic lipid profiles, and triggers thrombosis while also damaging the artery wall and causing endothelial dysfunction and atherosclerosis [13,14]. Expanded atheroma and low fibrous volume [15], further plaque bleeding [16], and increased inflammation and tissue death [17] have all been found in smokers' plaques. These changes result in a plaque composition more prone to rupture, leading to cardiovascular events. Long-term cigarette exposure in young people is linked to a two-fold increased risk of developing catastrophic carotid atherosclerotic plaques compared to non-smokers [18]. The present study aimed to assess the associations between fat depots, nicotine dependence (ND), and the incidence and severity of CAS.

## Material and Methods

### Study setting and population

This case-control study was conducted at Al-Imamain Al-Kadhumain Medical City from January to August 2021. Sixty patients of both sexes with varying degrees of CAS were enrolled in the study, while those with chronic liver disease, type 1 diabetes mellitus, kidney failure, or stroke were excluded. A control group of 60 healthy volunteers of similar ages and genders was also included, consisting of blood donors and healthy individuals who accompanied the patients. Both the patients and the controls were heavy smokers with varying degrees of intensity.

The diagnosis and severity of CAS were determined by a specialist using Doppler ultrasound (DUS) with a 7-MHz linear array transducer. Gray-scale, Color Doppler, and Spectral DUS were performed with an angle of insonation ≤60º. Subjects in the control group had CAS<30%.

### Data collection

The study collected information on age, sex, height, weight, waist-hip ratio (WHR), waist circumference (WC), body mass index (BMI), and family history of CAD through direct interviewing. Clinical data, including comorbidities and lipid profiles, were gathered from hospital records. Lipid accumulation products (LAP) and visceral adiposity index (VAI) were calculated for all participants according to the following formula:

LAP=[waist circumference (cm) - 58] ×

× triglycerides (mmol/l).

VAI=(WC (cm)/ (39, 68+ (1.88*BMI)*(TG/1.03)*

*(1.31/HDL) for men and

VAI=(WC (cm)/ (36, 58+ (BMI *1.89) *(TG/0.81)*

*(1.52/HDL) for women.

### Smoking duration, intensity, and nicotine dependence

Data on smoking duration (in years) and smoking intensity (number of cigarettes/day) were collected from each participant. Nicotine dependence was calculated using the Fagerström Test for Nicotine Dependence (FTND) [19], which consists of six items with varying scores: 1-2 = low dependence, 3-4 = low to moderate dependence, 5-7 = moderate dependence, and 8+ = high dependence.

### Statistical analysis

Statistical analyses were performed using SPSS software version 25.0 (SPSS, Chicago). Continuous variables were tested for normality using the Shapiro-Wilk test and were presented as mean and standard deviation (SD) for normally distributed data, while non-normally distributed data were presented as median and range. The Student t-test was used to analyze normally distributed data, while the Mann-Whitney U test and Kruskal Wallis test were used to compare non-normally distributed data between two and three groups, respectively. Categorical variables were presented as numbers and percentages and analyzed using the Chi-square test. The diagnostic performance of VAI and LAP in distinguishing between CAS and controls was evaluated using receiver operating characteristic (ROC) curve analysis. Spearman's correlation test was used to explore the potential association between ND and other variables. A p-value less than 0.05 was considered statistically significant.

## Results

### Demographic characteristics

There were no significant differences in age, gender distribution, BMI, or waist circumference between patients and controls. Comorbidities, on the other hand, were much more common among patients than among controls. Diabetes, hypertension, and ischemic heart disease were all significantly more common in patients (23.3%, 40%, and 13.3%, respectively) than in controls (10%, 21.7%, and 3.3%, respectively).

With a highly significant difference, the median smoking duration in patients was 23.5 years (range 4-45 years) compared to 15 years (range 2-50 years) in controls. Moreover, patients smoked a median of 40 cigarettes per day (range of 4-120 cigarettes per day), significantly greater than controls (20 cigarettes/day, range 4-60 cigarettes per day). Furthermore, nearly half of the patients (46.67%) exhibited a high level of nicotine dependency, compared to only 26.67% of the controls, a highly significant difference as indicated in Table 1.

**Table 1:** Demographic data of the study population.

Variables	CAS patients (n=60)	Controls (n=60)	P-value
**Age, years**			
Mean±SD	54.8±8.26	54.72±7.42	0.954
Range	42-65	40-65	
**Gender**			
Males	47 (78.3%)	46 (76.7%)	0.827
Female	13 (21.7%)	14 (23.3%)	
**Body mass index, kg/m^2^**			
Mean±SD	25.34±3.8	24.6±2.88	0.231
Range	19.2-36.5	21.6-34.6	
**Waist circumference, cm**			
Mean±SD	88.83±10.33	86.49±9.66	0.202
Range	70.4-112.0	67.6-109.3	
**Smoking duration, years**			
Mean±SD	24.12±10.15	17.12±10.8	<0.001
Median	23.5	15.0	
Range	4-45	2-50	
**Number of cigarettes/day**			
Mean±SD	33.85±15.43	27.5±21.9	0.009
Median	40.0	20.0	
Range	4-120	4-60	
**FTND**			
Low dependence	7 (11.67%)	13 (21.67%)	0.005
Low-moderate dependence	21 (35%)	19 (31.67%)	
Moderate dependence	4 (6.67%)	12 (20%)	
High dependence	28 (46.67%)	16 (26.67%)	
Comorbidities*			
Present	27 (45%)	16 (26.7%)	0.036
Diabetes mellitus	14 (23.3%)	6 (10%)	0.050
Hypertension	24 (40%)	13 (21.7%)	0.030
Ischemic heart disease	8 (13.3%)	2 (3.3%)	0.048

* – Many patients had more than one comorbidities; CAS – carotid artery stenosis; FTND – Fagerstrom Test for Nicotine Dependence.

### Lipid profile and related data

Except for vLDL, all lipid profile variables were significantly higher in patients than in controls (Table 2). The median VAI and LAP of patients were 3.92 and 32.83, respectively, significantly greater than those of controls (1.48 and 21.15, respectively).

**Table 2:** Lipid profile and related data of the study population.

Variable	CAS patients (n=60)	Controls (n=60)	P-value
**Total cholesterol level, mg/dL**			
Mean±SD	174.02±52.5	119.78±35.24	<0.001
Median	179.5	119	
Range	63.9-298	63.9-227	
**Triglyceride level, mg/dL**			
Mean±SD	151.84±64.33	94.47±57.95	<0.001
Median	152.0	89.0	
Range	42-360	42-360	
**HDL level, mg/dL**			
Mean±SD	33.91±1436	56.37±27.24	<0.001
Median	32.0	54.0	
Range	21-98	21-116.1	
**LDL level, mg/dL**			
Mean±SD	101.99±42.8	78.59±36.16	0.012
Median	85.0	76.0	
Range	47.7-198	20.11-198	
**vLDL level, mg/dL**			
Mean±SD	28.42±11.94	26.57±8.22	0.860
Median	22.9	22.9	
Range	15.37-71.95	15.37-44.8	
**Visceral adiposity index**			
Mean±SD	4.09±2.12	1.94±1.75	<0.001
Median	3.92	1.48	
Range	0.32-10.87	0.32-8.06	
**Lipid accumulation product**			
Mean±SD	38.97±24.93	26.42±18.68	0.001
Median	32.83	21.15	
Range	9.29-112.49	7.07-100.03	

CAS – carotid artery stenosis; HDL – high-density lipoprotein; LDL – low-density lipoprotein; vLDL – very low-density lipoprotein.

### Diagnostic value of VAI and LAP in detecting CAS

**Figure 1:**
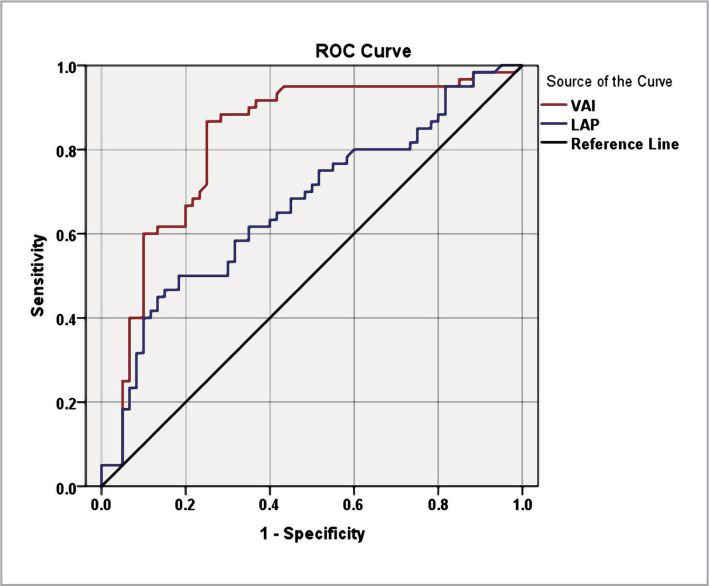
Receiver operating characteristic curve for visceral adiposity index and lipid accumulation products with carotid artery stenosis.

The sensitivity and specificity of VAI and LAP in detecting CAS were evaluated using the receiver operating characteristic (ROC) curve. The VAI showed an area under the curve (AUC) of 0.823 (95% confidence interval [CI]: 0.743-0.902, p < 0.001). At a cut-off value of VAI = 2.48, the sensitivity and specificity of the test were both 75%. The LAP had an AUC of 0.668 (95% CI: 0.571-0.765) with a p-value of 0.001. At a cut-off value of LAP = 26.75, the sensitivity and specificity of the test were 62% and 65%, respectively (Figure 1).

### Severity of carotid artery stenosis

**Figure 2:**
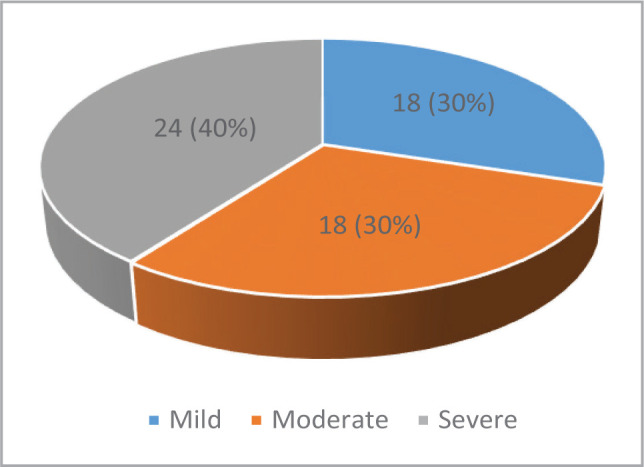
Distribution of patients according to the CAS severity.

According to the Doppler criteria for ICA stenosis diagnosis, eighteen patients (30%) had a mild degree of stenosis, another eighteen (30%) had a moderate degree of stenosis, and the other 24 patients (40%) had a severe degree of stenosis (Figure 2).

### Relationship between demographic characteristics and CAS severity

BMI and WC, but not age or concomitant diseases, were significantly associated with CAS severity (p=0.002 and 0.025, respectively). Moreover, smoking duration was associated with CAS severity, although cigarette count per day and FTND were not (Table 3).

**Table 3:** Relationship between demographic data and CAS severity.

Variable	CAS severity			P-value
	Mild (n=18)	Moderate (n=18)	Severe (n=24)	
**Age, years**				
Mean±SD	54.72±7.95	54.72±8.61	54.92±8.59	0.966
Range	43-65	43-65	42-65	
**BMI, Kg/m^2^**				
Mean±SD	23.32±2.28	24.76±2.46	27.29±4.62	0.002
Range	19.2-28	20.8-29.3	21.8-36.5	
**WC, cm**				
Mean±SD	83.72±6.81	89.27±7.67	92.33±12.74	0.025
Range	71.4-96.5	74.5-102.4	70.4-112	
**Smoking duration, years**				
Mean±SD	19.72±11.08^a^	24.33±8.48^ab^	27.25±9.74^b^	0.048
Median	17.0	23.0	26.5	
Range	5-43	10-40	4-45	
**No. of cigarettes/day**				
Mean±SD	29.78±17.66	31.22±16.51	38.88±11.59	0.151
Median	32.5	35	40.0	
Range	4-60	4-60	10-60	
**FTND**				
Low dependence	3 (16.67%)	4 (22.22%)	0 (0%)	0.077
Low-moderate dependence	7 (38.89%)	4 (22.22%)	10 (41.67%)	
Moderate dependence	4 (22.22%)	0 (0%)	0 (0%)	
High dependence	4 (22.22%)	10 (56.56%)	14 (58.33%)	
Co-morbid disease*				
Yes	11 (61.11%)	7 (38.89%)	9 (37.5%)	0.259
DM	6 (33.33%)	4 (22.22%)	4 (16.67%)	0.446
HT	9 (50%)	7 (38.89%)	8 (33.33%)	0.548
IHD	4 (22.22%)	2 (11.11%)	2 (8.33%)	0.401

Different small letters indicate Different small letters ^(a, ab, b)^ indicate significant differences significant differences. CAS – carotid artery stenosis; WC – waist circumference; FTND – fagerstrom test for nicotine ddependence; DM – diabetes mellitus; HT – hypertension; IHD – ischemic heart disease.

As shown in Table 4, there was a significant correlation between CAS severity and an increase in TG level, VAI, or LAP (p = 0.032, p = 0.007, and p = 0.013, respectively).

**Table 4:** Relationship between lipid profile and related data and CAS severity.

Variable	CAS severity			P-value
	Mild (n=18)	Moderate (n=18)	Severe (n=24)	
**TC level, mg/dL**				
Mean±SD	152.84±45.84	170.6±47.47	192.46±56.01	0.071
Median	154.0	168.0	203.0	
Range	63.9-229	98.8-233	116-298	
**TG level, mg/dL**				
Mean±SD	123.94±46.85^a^	149.46±69.53^ab^	174.54±63.67^b^	0.032
Median	109.0	120.5	166.5	
Range	42-212	65.9-360	77-320	
**HDL level, mg/dL**				
Mean±SD	38.8±19.69	33.08±15.2	30.87±6.52	0.178
Median	34.7	30.5	32.0	
Range	21-98	22.8-89	21-43	
**LDL level, mg/dL**				
Mean±SD	97.62±42.35	107.88±50.32	100.85±38.19	0.798
Median	83.0	87.0	88.5	
Range	53.5-198	47.7-198	47.7-169.3	
**vLDL level, mg/dL**				
Mean±SD	24.74±8.26	30.31±13.41	29.76±12.93	0.392
Median	21.86	24.12	30.47	
Range	15.37-43	15.95-71.95	15.37-71.95	
**VAI**				
Mean±SD	3.03±1.77^a^	3.84±1.86^ab^	5.08±2.2^b^	0.007
Median	2.57	3.41	4.81	
Range	0.32-6.58	0.54-7.3	1.69-10.87	
**LAP**				
Mean±SD	24.91±12.41^a^	40.3±20.52^ab^	48.52±30.3^b^	0.013
Median	22.8	38.52	46.91	
Range	9.29-49.93	13.09-82.34	10.9-112.49	

Different small letters ^(a, ab, b)^ indicate significant differences. CAS – carotid artery stenosis, TC = total cholesterol; TG – triglyceride; HDL – high-density lipoprotein; LDL – low-density lipoprotein; vLDL – very low-density lipoprotein; VAI – visceral adiposity index; ALP – lipid accumulation product.

## Discussion

In this study, patients with CAS were found to have significantly higher smoking intensity and duration, as well as higher FTND scores, compared to controls. These findings are consistent with previous studies, such as Babiker et al. [20], who investigated how smoking affected carotid artery hemodynamics in Saudi Arabia. They found a significant correlation between smoking frequency and plaque presence (p < 0.001). Their data revealed a strong linear relationship between smoking duration and CAS severity, which increased by 0.34% annually, as well as between smoking frequency and carotid narrowing severity, which increased by 0.31% per unit of frequency. Tell et al. [21] also found that longer smoking periods were consistently associated with an increased risk of external CAS after adjusting for all potential confounders. Smoking increases the risk of stroke in several ways, some of which are not fully understood. Short-term effects include the generation of thrombi in atherosclerotic arteries, and long-term effects include the development of atherosclerotic stenosis [22]. According to FTND, approximately half of the patients with CAS in our study had high dependence, compared to about one-fourth of controls.

Zhu et al. [23] investigated the role of ND as a risk of atherosclerosis in long-term cigarette smokers in a nested case-control study with 166 patients and 286 controls. Smoking 1 min after awakening has a higher risk of developing aortic atherosclerosis than smoking 5 minutes after awakening (OR = 0.24 (95% CI 0.08, 0.69). Maroules et al. [24] also found that a later time to first smoke was associated with a lower risk of aortic atherosclerosis, an independent predictor of cardiovascular disease events. [24]. In addition, smoking the first cigarette soon after waking up was significantly associated with lower HDL levels, a lower LDL/HDL ratio, and high cholesterol (> 200 mg/dL), although the mechanism underlying this association is not yet fully understood. Oral nicotine has been shown to increase LDL and decrease the HDL/total cholesterol ratio by impairing the clearance of LDL from the plasma [25].

In the present study, VAI and LAP indices were significantly higher in CAS patients than in controls. Son et al. [26] assessed the relationship between the dynamic change of VAI and the risk of carotid plaque in 23522 Chinese participants. They calculated VAI at baseline and follow-up and found a significant positive correlation between VAI and carotid plaque risk (HR, 1.53; 95% CI, 1.48-1.59 [P 0.001]) in a nonlinear dose-response pattern. Interestingly, they observed that men had a much higher risk of developing carotid plaque than women.

Previous research has demonstrated a direct connection between cardiovascular disease, abdominal obesity, and adipose tissue dysfunction [27,28]. Abdominal fat accumulation may independently increase the risk of cardiovascular disease, and adipocytes may mechanically facilitate the increased arterial stiffness associated with obesity [29]. In a retrospective study, VAI was found to be 39% more prevalent in patients with CAS and was significantly higher in these patients [30]. Leptin, adiponectin, interleukin-6, and tumor necrosis factor are only a few cytokines and bioactive mediators released by adipose tissue, an active endocrine and paracrine organ that may affect blood flow and encourage atherosclerosis [31]. Xia et al. [32] showed that VAI, a simple clinical index reflecting visceral fat mass in clinical practice, comprises age, WC, triglycerides, HDL-C, and BMI. The inflammatory atherothrombotic pathway is crucial to the development of atherosclerosis, and visceral adipose tissue plays a significant role in this pathway [33]. High levels of visceral fat tissue are associated with increased secretion of inflammatory cytokines, such as interleukin-6, tumor necrosis factor-alpha, and plasminogen activator inhibitor type-1, and decreased secretion of the protective and anti-inflammatory adiponectin protein [34]. Studies have shown that compared to subcutaneous fat, visceral fat tissue is more pathogenic and contributes more significantly to cardiometabolic complications [35].

In the present study, BMI and WC were significantly associated with CAS severity. Ferreira et al. [8] also reported a link between obesity and other carotid atherosclerotic plaque characteristics, showing that carotid plaque instability was linked to overall obesity and an increase in fat mass. Similarly, Salari et al. [36] found that moderate to severe atherosclerosis affected 62% of the adult population of northern Iran and was correlated with obesity indices. Numerous studies have demonstrated a direct relationship between some obesity indices and atherosclerosis. For instance, Pokharel et al. [37] revealed a correlation between BMI and WC and subclinical atherosclerosis, while Oh et al. [38] demonstrated a positive correlation between WHR and an elevated risk for coronary artery calcification progression. Increased endothelial dysfunction, inflammation, and associated cardiovascular risk factors may contribute to the higher frequency of atherogenic events in individuals with high BMI [39].

The length of smoking was significantly correlated with the severity of CAS, consistent with Whisnant et al. [40], who looked at 752 patients to assess the degree of carotid atherosclerosis and found that the number of years of smoked cigarettes was the most significant independent predictor of the presence of severe carotid atherosclerosis. Another study using multiple linear regression found that age, hypertension, and pack-years of smoking were positively correlated with the extent of extracranial CAS in patients who underwent noninvasive assessment of extracranial CAS by duplex ultrasonography [41]. In the present study, the VAI and LAP were significantly associated with the severity of CAS. In a study conducted in Hungary, Bagyura et al. [42] assessed the relationship between VAI and the severity of atherosclerosis in asymptomatic subjects. The study discovered that compared to men without CAS, those with higher CAS had VAIs significantly higher. Even after adjusting for a number of factors (OR 3.21, 95% CI: 1.16-8.85, p = 0.024), the risk of CAS was significantly higher in the upper VAI tertiles compared to the lowest tertile (OR 3.41, 95% CI: 1.4-8.31, p = 0.007). Park et al. [43] evaluated more than 33,000 patients from the Korean population, and both sexes displayed a strong correlation between VAI and CAS. Randrianarisoa et al. [44] found a significant association between VAI and carotid intima-media thickness, a marker of subclinical atherosclerosis, along with age, smoking, and male sex. This finding is consistent with Kouli et al. [45], who found that VAI is independently associated with elevated 10-year CVD risk, particularly in men. There is no precise cut-off point for VAI that can be used to distinguish between healthy and unhealthy visceral adiposity. Some researchers use quartiles as the cut-off value for the analysis, while others use tertiles [46,47]. LAP was strongly linked to the atherogenic profile of lipoprotein subfractions in a Brazilian study [48]. Additionally, LAP was negatively correlated with HDL-C and positively correlated with TC, glucose, insulin, HOMA-IR, and non-esterified fatty acids values. This explains how these indices and the severity of CAS are related.

## Conclusion

Our study found that CAS patients had a higher prevalence of prolonged smoking duration, increased intensity, and higher ND compared to healthy individuals. Additionally, VAI and LAP, indicators of visceral and central obesity, were elevated in CAS patients and demonstrated excellent diagnostic value in detecting CAS. Furthermore, our findings suggest that VAI and LAP are strong predictors of the development of severe CAS. Preventive measures aimed at reducing the burden of CAS should emphasize the importance of smoking cessation and increase public awareness of the significant dangers associated with smoking (duration and intensity).

## Data Availability

Further data is available from the corresponding author upon reasonable request.

## Personal thanks

We express our gratitude to Ms. Jasim E.A. and Ms. Ahmed I.W. from the Medical Research Unit, College of Medicine, Al-Nahrain University, for their valuable contribution and assistance in laboratory techniques.
